# Supplementary material for: Complex structure of cytochrome c–cytochrome c oxidase reveals a novel protein–protein interaction mode
Source: EMBO J. 2016 Dec 15;36(3):291–300. doi: 10.15252/embj.201695021 (PMC5286356; doi:10.15252/embj.201695021)
Supplement: Supplementary file 1 — Appendix [file EMBJ-36-291-s001.pdf]

Appendix Information

Complex structure of cytochrome *c*–cytochrome *c* oxidase shows a novel protein-protein interaction mode

Satoru Shimada, Kyoko Shinzawa-Itoh*, Junpei Baba, Shimpei Aoe, Atsuhiro Shimada, Eiki Yamashita, Jiyoung Kang, Masaru Tateno, Shinya Yoshikawa, Tomitake Tsukihara*

*Corresponding author. E-mail: shinzawa@sci.u-hyogo.ac.jp (K.S.-I.)

tsuki@protein.osaka-u.ac.jp (T.T.)

Table of contents:

Appendix Figures S1-9

Appendix Tables S1-3

Appendix References

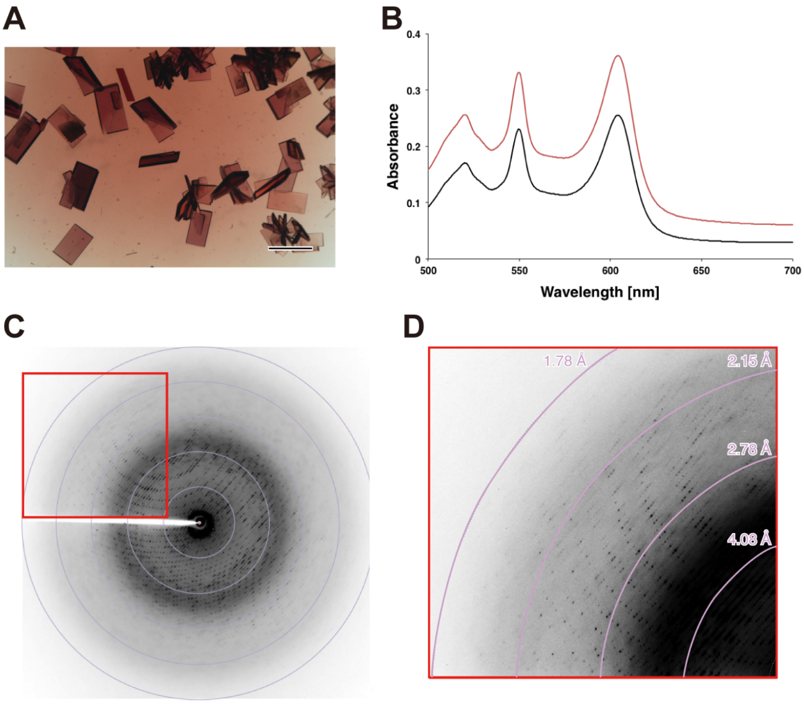

Appendix Figure S1. Crystals, absorption spectra, and diffraction image of mammalian Cyt.*c*−C*c*O complex.

A Rectangular-plate crystals of Cyt.*c*−C*c*O complex grown by the batch-wise method from a solution containing Cyt.*c*/C*c*O at molar ratio of 1.2. Scale bar indicates 1.0 mm.

B Dithionite-reduced spectra of the solution before crystallization (black) and of the resultant crystals (red). Reductions of heme *c* and heme *a* are indicated by absorbance peaks at 550 nm and 604 nm, respectively.

C X-ray diffraction image from a Cyt.*c*-C*c*O complex crystal.

D Close-up view of the region indicated the box in panel (C).

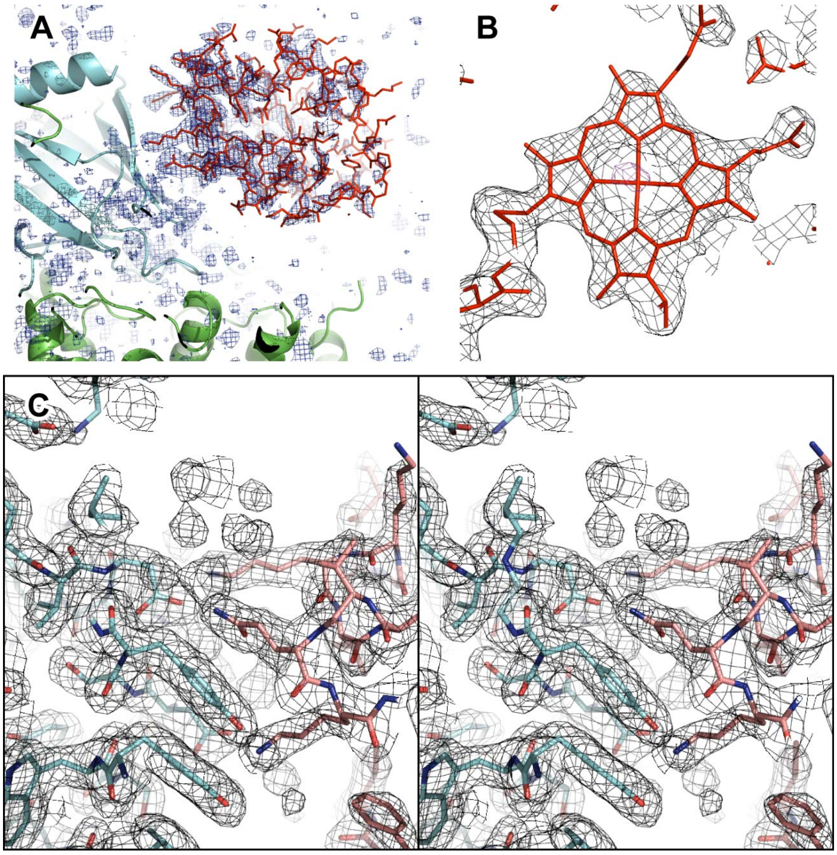

Appendix Figure S2. Electron density maps of the Cyt.*c* and the interface between Cyt.c and CcO.

A F_O–F_C difference electron-density map, calculated with the phase after molecular replacement using the C*c*O molecule, is represented as blue mesh contoured at the 2.5 σ level. The Cyt.*c* model is shown as red sticks for clarity. Subunit II and other subunits of C*c*O are shown as light blue and green ribbons, respectively.

B Close-up views of the 2*Fo*–*Fc* map for the heme and the anomalous difference map for iron atoms, represented as gray mesh at the 1.0 σ level and purple mesh at the 3.5 σ level, respectively. Bonds are colored in red.

C The Cyt.*c*–C*c*O interface is shown by a stereoscopic pair of 2*Fo*–*Fc* maps drawn at the 1.0 σ level. Stick models of Cyt.*c* and C*c*O are represented as in Fig 3C.

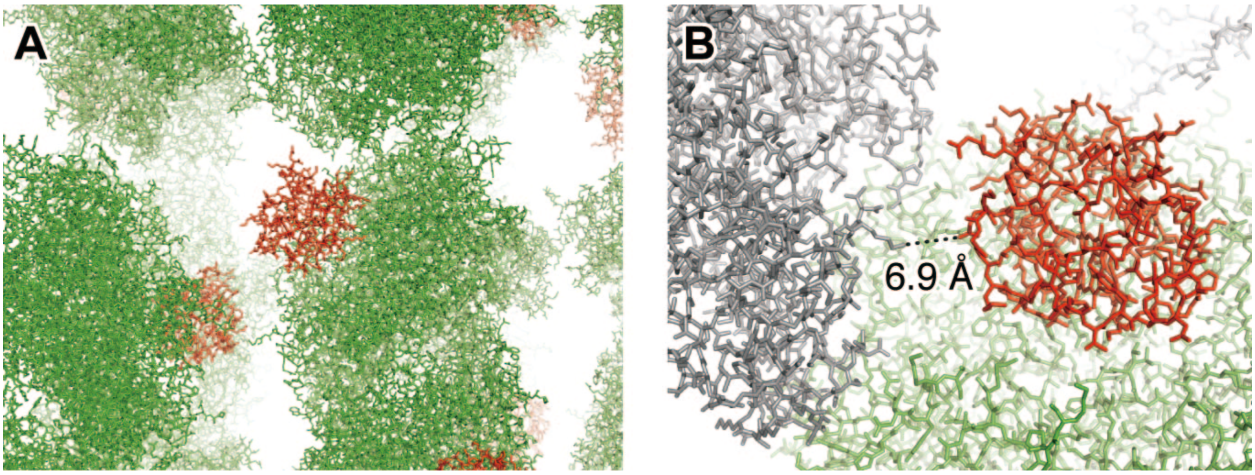

Appendix Figure S3. Crystal packing of the Cyt.*c*–C*c*O complex.

A Cyt.*c*–C*c*O complex in the lattice of the *P*21 crystal, represented as sticks (green: C*c*O; red: Cyt.*c*).

B Close-up view of close contacts between Cyt.*c* and a symmetry-related Cyt.*c*–C*c*O complex, colored in grey. Dotted line indicates the distance of 6.9 Å between side-chain atoms of Glu21 of Cyt.*c* and Lys108 of subunit Va of C*c*O related by a crystallographic symmetry. This is the shortest distance between the Cyt.*c* of a Cyt.*c*–C*c*O complex and any Cyt.*c*–C*c*O complex related by a crystallographic symmetry operation.

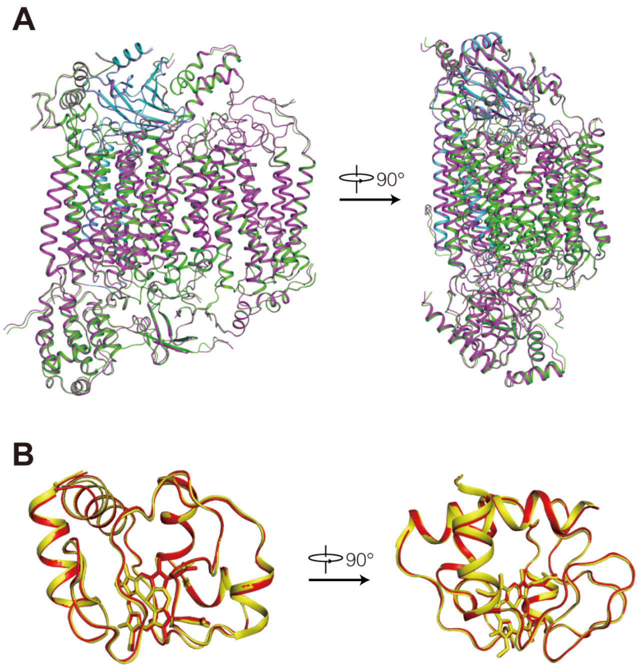

Appendix Figure S4. Structural comparison of individual proteins between the Cyt.*c*–C*c*O complex and free C*c*O or Cyt.*c*.

A Superimposition of the main chains of C*c*O in the Cyt.*c*–C*c*O complex (green) and free C*c*O (magenta, PDB 5B1A). R.m.s.d. for Ca atoms is 0.47 Å .

B Superimposition of the main chains of Cyt.*c* in the Cyt.*c*–C*c*O complex (red) and free Cyt.*c* (yellow, PDB 1HRC). R.m.s.d. for Ca atoms is 0.41 Å .

Both proteins in the complex are not significantly different from their individual states in their main chain structures.

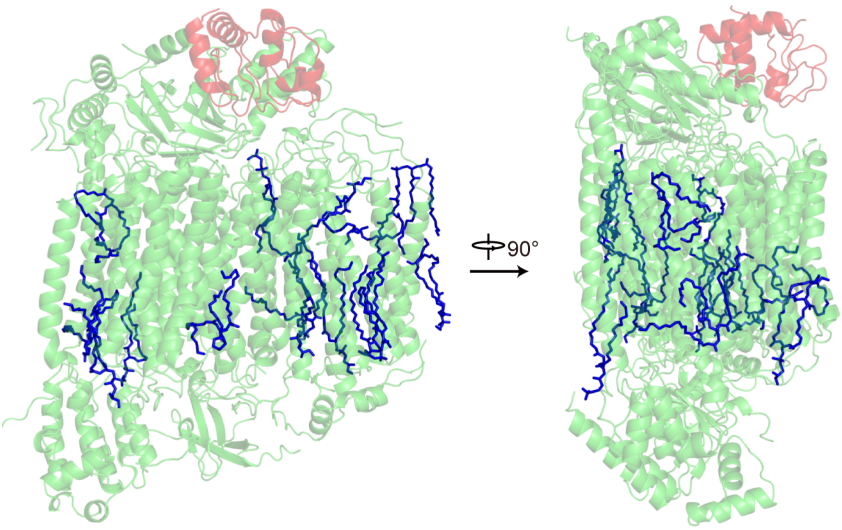

Appendix Figure S5. Arrangement of lipid molecules in the Cyt.*c*−C*c*O complex.

A total of eleven lipid molecules (one phosphatidylethanolamine, four phosphatidylglycerol, two cardiolipin, one phosphatidyl-choline, three triacylglycerol) are shown as blue sticks. No phospholipid is close to Cyt.*c*.

A

```
Human        1    MAHAAQVGLQDATSPIMEELITFHDHALMIIFLICFLVLYALFLTLTTKLTNTNISDAQE    60
Bovine       1    MAYPMQLGFQDATSPIMEELLHFHDHTLMIVFLISSLVLYIISLMLTTKLTHTSTMDAQE    60
Pig          1    MAYPFQLGFQDATSPIMEELLHFHDHTLMIVFLISSLVLYIISLMLTTKLTHTSTMDAQE    60
Mouse        1    MAYPFQLGLQDATSPIMEELMNFHDHTLMIVFLISSLVLYIISLMLTTKLTHTSTMDAQE    60
Chicken      1    MANHSQLGFQDASSPIMEELVEFHDHALMVALAICSLVLYLLTLMLMEKLSS-NTVDAQE    59
Zebrafish    1    MAHPAQLGFQDAASPVMEELLCFHDHALMIVFLISTLVLYIIIAMVSTKLTNKFILDSQE    60
                  **   *:*:***:**:****: ****:**: : *. **** :   :  **:     *:**

                                                              105
Human       61    METVWTILPAIILVLIALPSLRILYMTDEVNDPSLTIKSIGHQWYWTYEYTDYGGLIFNS   120
Bovine      61    VETIWTILPAIILILIALPSLRILYMMDEINNPSLTVKTMGHQWYWSYEYTDYEDLSFDS   120
Pig         61    VETIWTILPAIILILIALPSLRILYMMDEINNPALTVKTMGHQWYWSYEYTDYEDLTFDS   120
Mouse       61    VETIWTILPAVILIMIALPSLRILYMMDEINNPVLTVKTMGHQWYWSYEYTDYEDLCFDS   120
Chicken     60    VELIWTILPAIVLVLLALPSLQILYMMDEIDEPDLTLKAIGHQWYWTYEYTDFKDLSFDS   119
Zebrafish   61    IEIVWTVLPAIILILIALPSLRILYLMDEINDPHVTIKAVGHQWYWSYEYTDYENLEFDS   120
                  :* :**:***::*:::*****:***: **:::* :*:*::******:*****:  * *:*

Human      121    YMLPPLFLEPGDLRLLDVDNRVVLPIEAPIRMMITSQDVLHSWAVPTLGLKTDAIPGRLN   180
Bovine     121    YMIPTSELKPGELRLLEVDNRVVLPMEMTIRMLVSSEDVLHSWAVPSLGLKTDAIPGRLN   180
Pig        121    YMIPTSDLKPGEMRLLEVDNRVVLPMEMTIRMLVSSEDVLHSWAVPSLGLKTDAIPGRLN   180
Mouse      121    YMIPTNDLKPGELRLLEVDNRVVLPMELPIRMLISSEDVLHSWAVPSLGLKTDAIPGRLN   180
Chicken    120    YMTPTTDLPLGHFRLLEVDHRIVIPMESPIRVIITADDVLHSWAVPALGVKTDAIPGRLN   179
Zebrafish  121    YMVPTQDLTPGGFRLLETDHRMVVPKESPIRILVSAEDVLHSWAVPSLGIKMDAVPGRLN   180
                  ** *   *  * :***:.*.*:*:* *  **::::::*********:**:* **:*****

                                            207
Human      181    QTTFTATRPGVYYGQCSEICGANHSFMPIVLELIPLKIFEMGPVFTL---             227
Bovine     181    QTTLMSSRPGLYYGQCSEICGSNHSFMPIVLELVPLKYFEKWSASML---             227
Pig        181    QTTLMSTRPGLYYGQCSEICGSNHSFMPIVLELVPLKYFEKWSTSMLT--             228
Mouse      181    QATVTSNRPGLFYGQCSEICGSNHSFMPIVLEMVPLKYFENWSASMI---             227
Chicken    180    QTSFITTRPGVFYGQCSEICGANHSYMPIVVESTPLKHFEAWSSLLSS--             227
Zebrafish  181    QTAFIVSRPGVFYGQCSEICGANHSFMPIVVEAVPLEFFENWSSAMLEDA             230
                  *::.  .***::*********:***:****:*  **: **
```

B

```
                               1314
Human        1    MGDVEKGKKIFIMKCSQCHTVEKGGKHKTGPNLHGLFGRKTGQAPGYSYTAANKNKGIIW    60
Horse        1    MGDVEKGKKIFVQKCAQCHTVEKGGKHKTGPNLHGLFGRKTGQAPGFTYTDANKNKGITW    60
Bovine       1    MGDVEKGKKIFVQKCAQCHTVEKGGKHKTGPNLHGLFGRKTGQAPGFSYTDANKNKGITW    60
Rabit        1    MGDVEKGKKIFVQKCAQCHTVEKGGKHKTGPNLHGLFGRKTGQAVGFSYTDANKNKGITW    60
Chicken      1    MGDIEKGKKIFVQKCSQCHTVEKGGKHKTGPNLHGLFGRKTGQAEGFSYTDANKNKGITW    60
Bonito       1    MGDVAKGKKTFVQKCAQCHTVENGGKHKVGPNLWGLFGRKTGQAEGYSYTDANKSKGIVW    60
                  ***: **** *: **:******:*****.**** ********** *::** ***.*** *

Human       61    GEDTLMEYLENPKKYIPGTKMIFVGIKKKEERADLIAYLKKATNE                  105
Horse       61    KEETLMEYLENPKKYIPGTKMIFAGIKKKTEREDLIAYLKKATNE                  105
Bovine      61    GEETLMEYLENPKKYIPGTKMIFAGIKKKGEREDLIAYLKKATNE                  105
Rabit       61    GEDTLMEYLENPKKYIPGTKMIFAGIKKKDERADLIAYLKKATNE                  105
Chicken     61    GEDTLMEYLENPKKYIPGTKMIFAGIKKKSERVDLIAYLKDATSK                  105
Bonito      61    NENTLMEYLENPKKYIPGTKMIFAGIKKKGERQDLVAYLKSATS-                  104
                   *:********************.***** ** **:****.**.
```

Appendix Figure S6. Sequence alignments of vertebrate Cyt.*c* and C*c*O.

The sequences were aligned using Clustal Omega (Sievers *et al,* 2011). The residues included in the proposed ET pathway are indicated by red rectangles.

A Aligned sequences of subunit II of C*c*O (UniProt ID, Human - P00403; Bovine - P68530; Pig - P50667; Mouse - P00405; Chicken - P18944; Zebrafish - Q9MIY7).

B Aligned sequences of Cyt.*c* (UniProt ID, Human - P99999; Horse - P00004; Bovine - P62894; Rabit - P00008; Chicken - P67881; Bonito - P00025).

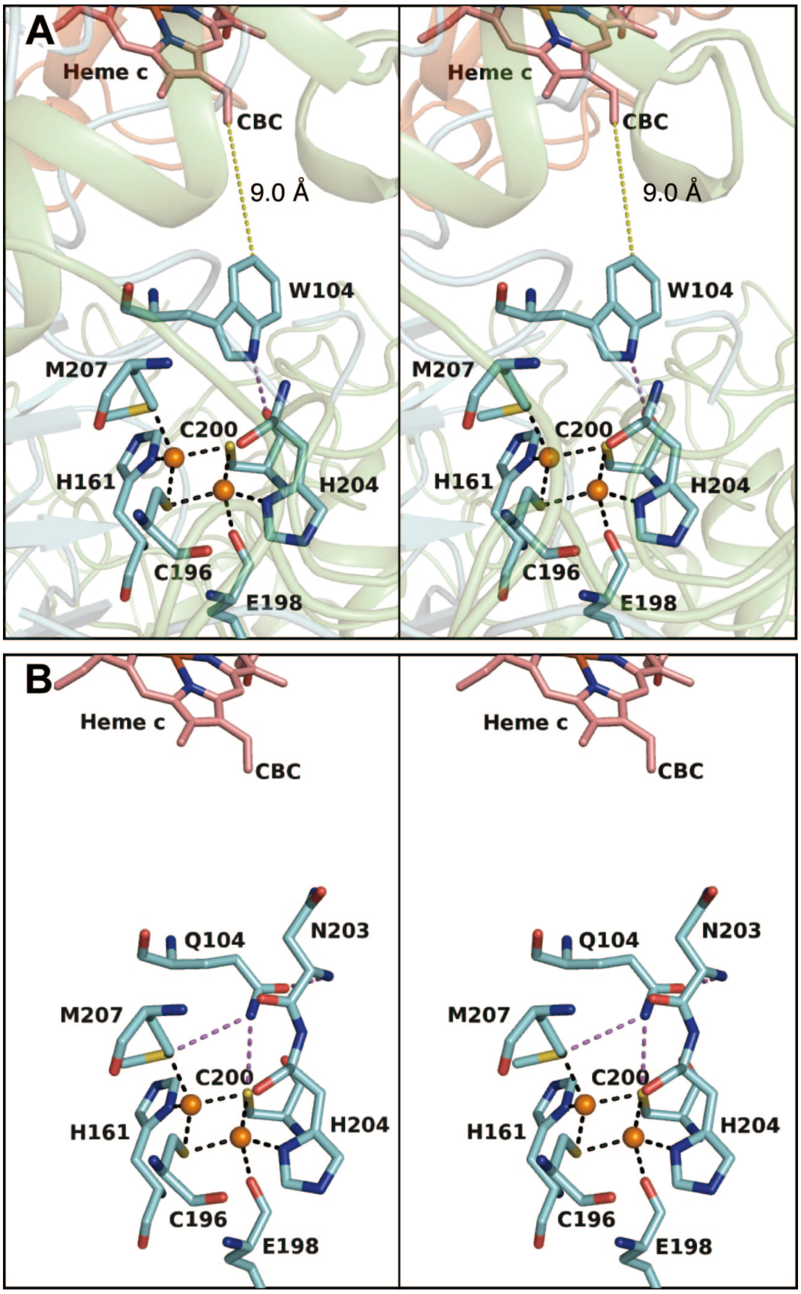

Appendix Figure S7. Stereoscopic drawings of spatial location of Trp^{104} of C*c*O subunit II in the docking interface and a predicted structure of the W104Q mutant.

Heme *c* of Cyt.*c*, and Tyr^{104} (or Gln^{104}), Cu_A and residues coordinating to copper ions of Cu_A of C*c*O subunit II are represented by stick models. Orange spheres represent copper ions of Cu<u>A</u>. Bonds are colored as in Fig 3C.

A Wild type.

B W104Q mutant.

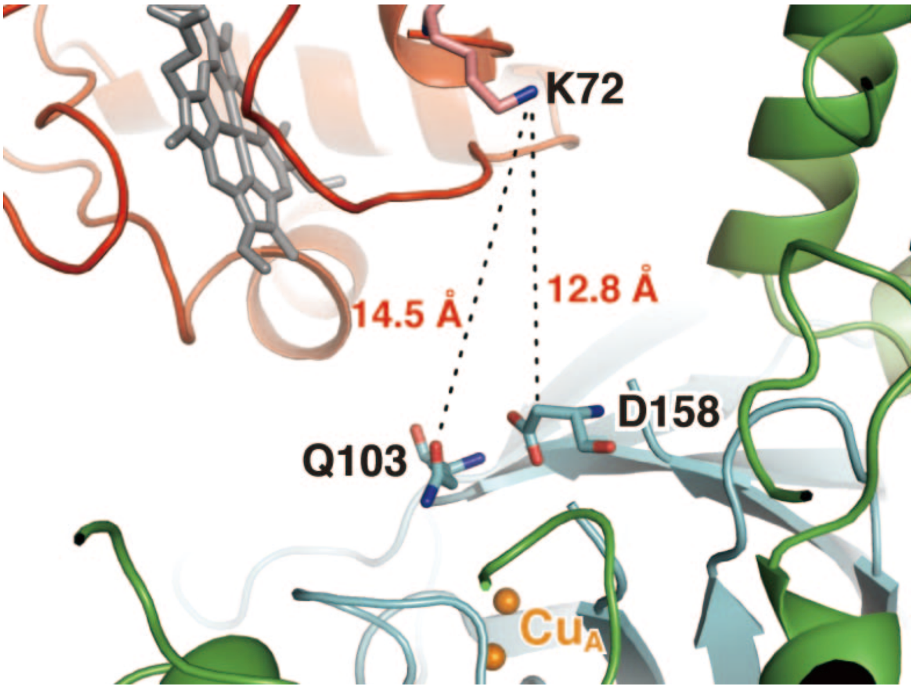

Appendix Figure S8. Arrangement of K72 of Cyt.*c*, and Q103 and D158 of C*c*O subunit II. The distances from K72 of Cyt.*c* to Q103 and D158 of subunit II of C*c*O in the crystal are 14.6 and 13.7 Å, respectively. Q103 and D158 of subunit II of C*c*O were predicted to interact with K72 of Cyt.*c* by structural simulation coupled and chemical modification studies (Ferguson-Miller et al, 1978; Roberts & Pique, 1999). Color representations are as in Fig 3C.

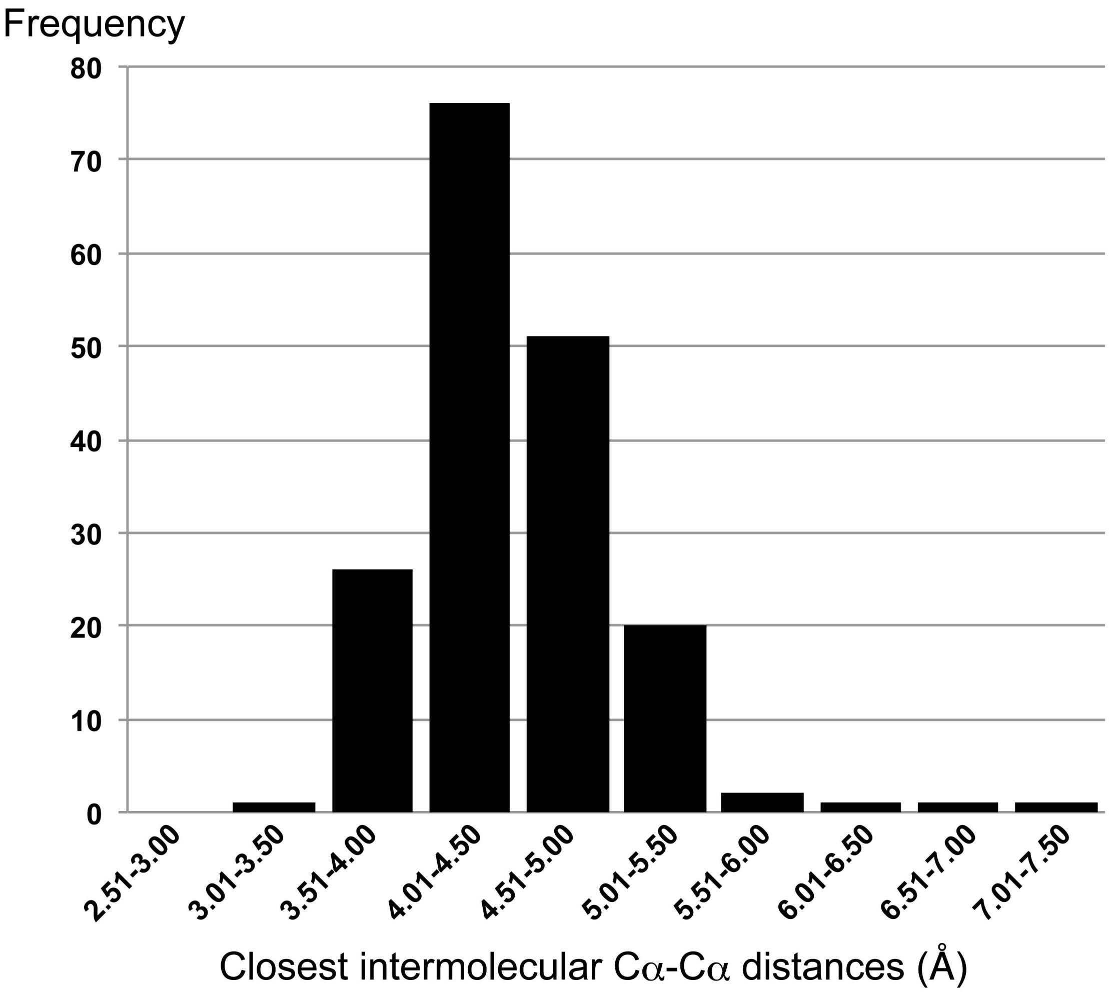

Appendix Figure S9. Histogram of closest inter-molecular Cα distances. Distances between Cα atoms of different proteins for 179 crystals of protein-protein complex compiled by Ahmed *et al* (Ahmed *et al*, 2011) were calculated. The bar graph shown statistics of the shortest distance in each crystal. The horizontal and the vertical axes of the graph indicate the distance and the frequency, respectively.

Appendix Table S1.   Number of waters in protein-protein interface

**Type of waters**	**Cyt.*c*–C*c*O**	**Cyt.*bc*$_1$–Cyt.*c***	**Cyt.*c*–C*c*P**
Non-bridging at Cyt.*c*	23	3	6
Non-bridging at the partner	19	13	13
Bridging	8	7	12
Non-interacting	14	2	4
All	64	25	35

Appendix Table S2. Averaged number of atoms closely contact with four types of waters (less than 3.5 Å)

Type of waters	Polar atoms	Non-polar atoms	Waters
Non-bridging at Cyt.*c*	1.3	1.2	1.4
Non-bridging at C*c*O	1.5	0.7	1.9
Bridging	2.0	0.9	1.6
Non-interacting	0.0	0.0	2.1

Appendix Table S3. The number of interaction between water and amino acid.

Amino acids	Waters at C*c*O	Amino acids	Waters at Cyt.*c*
Asp	12	Lys	16
Tyr	5	Gln	11
Glu	4	Ile	5
Asn	4	Glu	3
Gln	3	Thr	2
Ile	3	Val	2
Ser	3	Ala	1
Trp	2	Gly	1
His	2		
Met	2		
Ala	1		
Thr	1		

Appendix References

Ferguson-Miller S, Brautigan DL, Margoliash E (1978) Definition of cytochrome *c* binding domains by chemical modification III. Kinetics of reaction of carboxydinitrophenyl cytochrome *c* with cytochrome *c* oxidase. *J Biol Chem* 253: 149–159

Lyons JA, Aragao D, Slattery O, Pisliakov AV, Soulimane T, Caffrey M (2012) Structural insights into electron transfer in caa_3-type cytochrome oxidase. *Nature* 487: 514–518

Roberts VA, Pique ME (1999) Definition of the interaction domain for cytochrome *c* on cytochrome *c* oxidase: III. Prediction of the docked complex by a complete, systematic search. *J Biol Chem* 274: 38051–38060

Sievers F, Wilm A, Dineen DG, Gibson TJ, Karplus K, Li W, Lopez R, McWilliam H, Remmert M, Söding J, Thompson JD, Higgins D (2011) Fast, scalable generation of high-quality protein multiple sequence alignments using Clustal Omega. *Mol Syst Biol* 7: 539
