# Supplementary material for: Complex structure of cytochrome c–cytochrome c oxidase reveals a novel protein–protein interaction mode
Source: EMBO J. 2016 Dec 15;36(3):291–300. doi: 10.15252/embj.201695021 (PMC5286356; doi:10.15252/embj.201695021)
Supplement: Supplementary file 2 — Expanded View Figures PDF [file EMBJ-36-291-s002.pdf]

Expanded View Figures

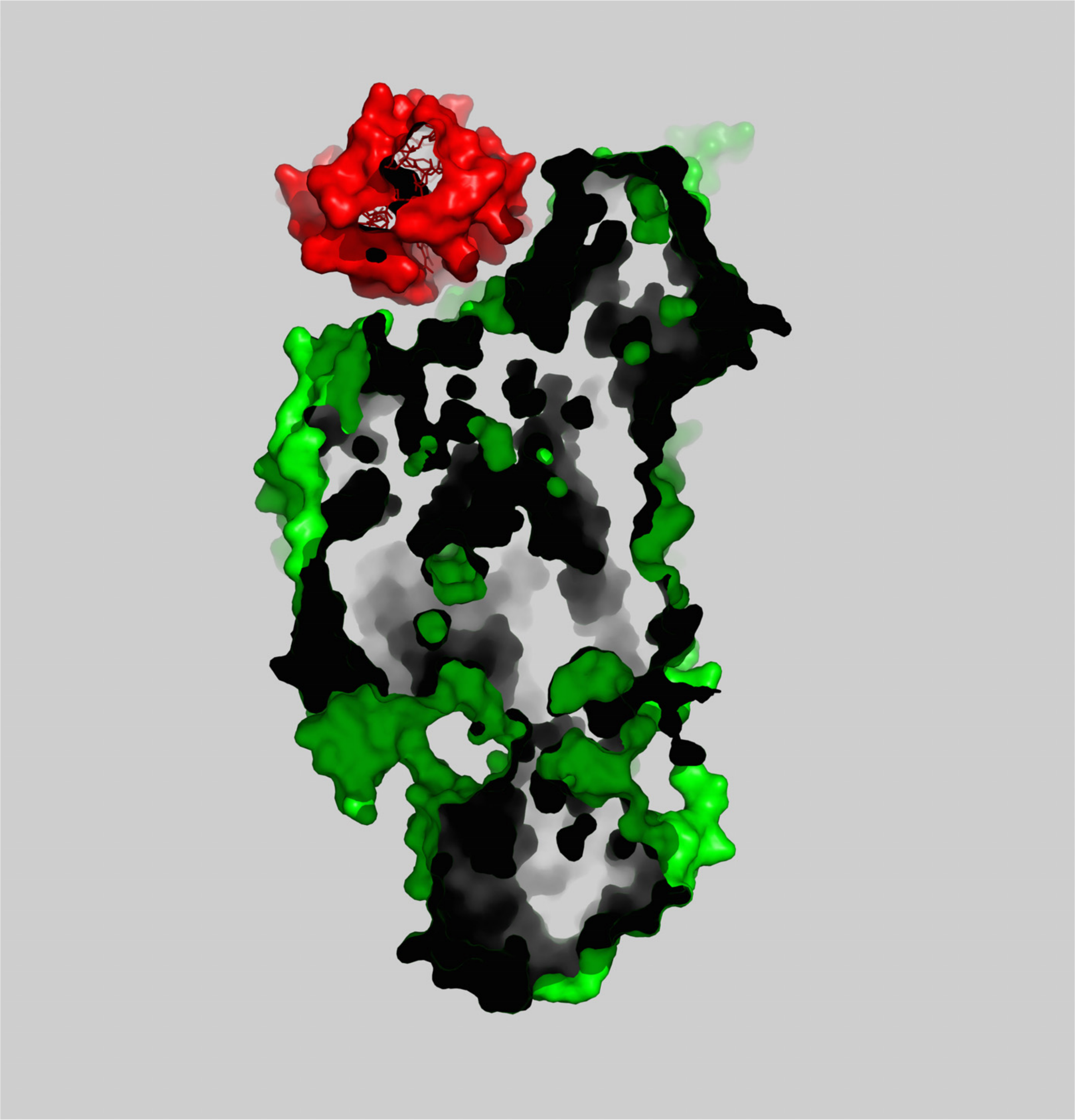

Figure EV1. A cross-section of surface structures of CcO and Cyt.*c*–CcO complex.
Cyt.*c* and CcO are shown as surface representation each colored in red and green, respectively. The cross-section of the surface representation indicates that Cyt.*c* fits closely with the concave surface.

© 2016 The Authors

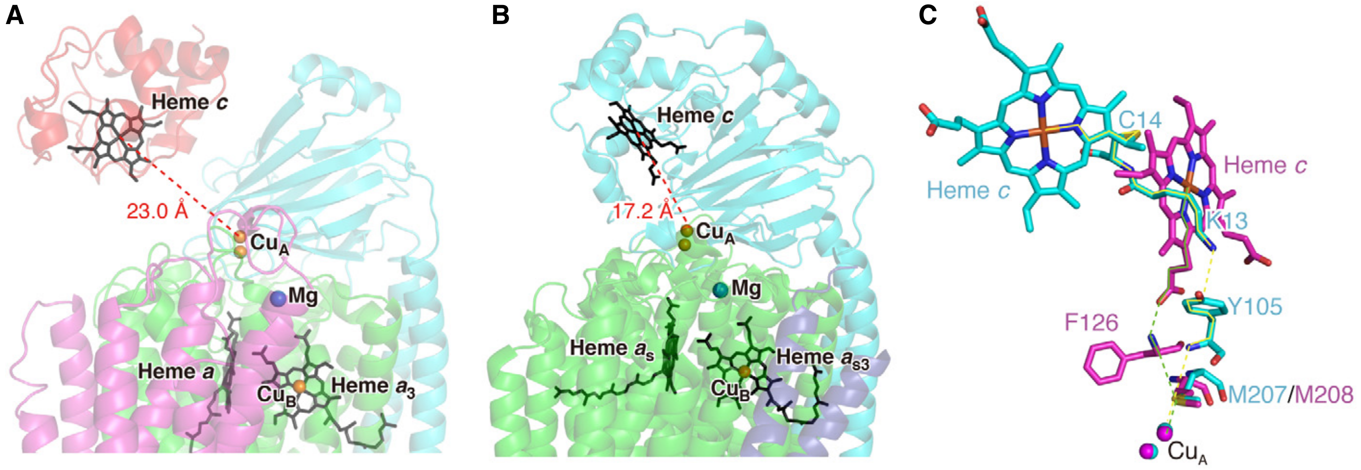

Figure EV2. Comparison of the Cyt.*c*–CcO complex with *caa*$_3$-type CcO (Lyons *et al*, 2012).

A, B Arrangement of redox cofactors in the Cyt.*c*–CcO complex (A) and *caa*$_3$-type CcO (B). Heme groups (*c*, *a*, *a*$_3$, *a*$_s$, and *a*$_{s3}$) are shown as black sticks. Copper and magnesium ions are represented by orange and blue spheres, respectively. Distances from iron of heme *c* to Cu$_A$ are indicated.

C Comparison of ET pathways from iron of heme *c* to Cu$_A$ between the Cyt.*c*–CcO complex (cyan sticks and yellow lines) and *caa*$_3$-type CcO (magenta sticks and green lines). Both structures are superposed with their Cu$_A$-containing domains. Solid and dashed lines represent through-bond and through-space processes, respectively.

 © 2016 The Authors

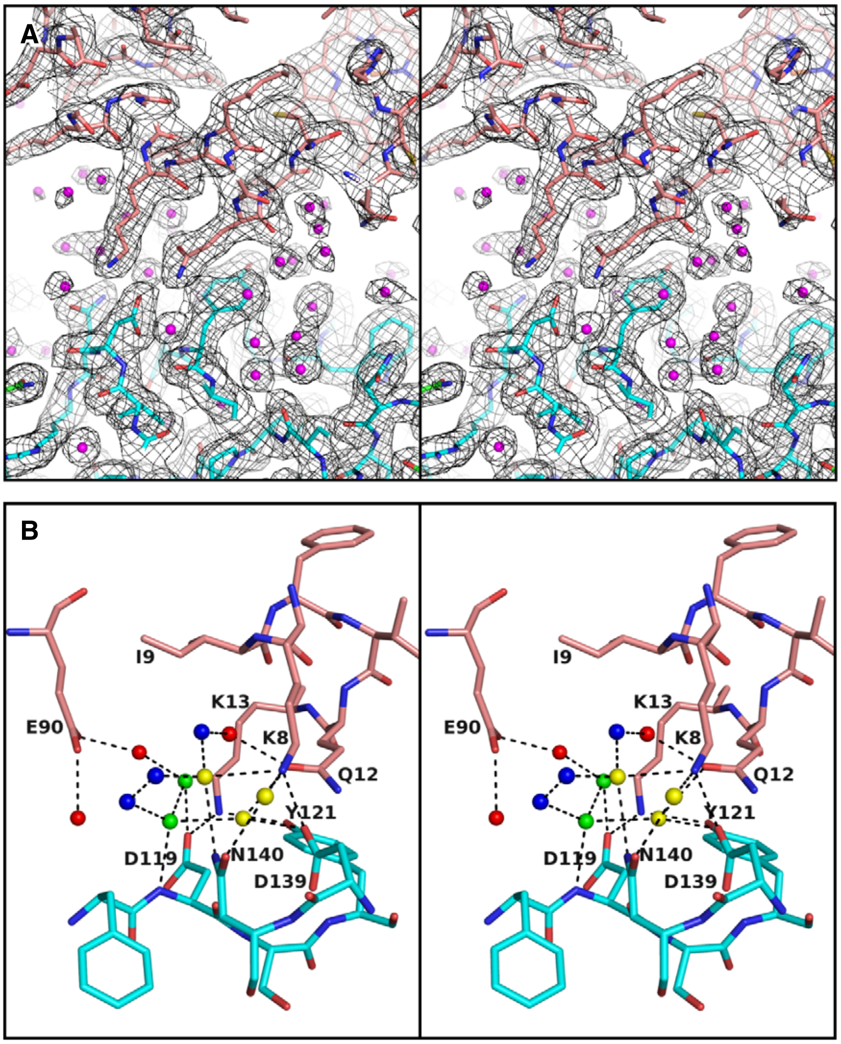

Figure EV3. Structure of waters around the direct interaction region between Cyt.*c* and C*c*O.
Stick models of Cyt.*c* and C*c*O are drawn in the same colors as in Fig 3C. Water is shown by a sphere.

A Stereoscopic view of the 2(F_o–F_c) map of the Cyt.*c*–C*c*O complex, drawn at the 1.0 σ level. Each water molecule is clearly assigned in the map.

B Stereoscopic view of water structure. Each water molecule is drawn in the same color as in Fig 4A. Hydrogen bond networks consisting of water molecules including non-interacting waters link Cyt.*c* and C*c*O.

© 2016 The Authors

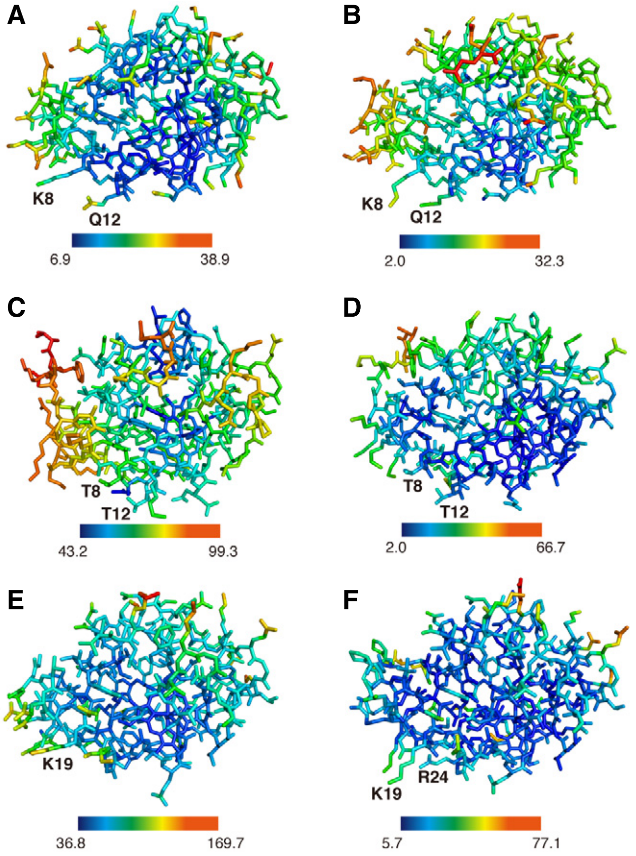

Figure EV4. Distribution of *B*-factors of Cyt.*c* molecules.

A–F Stick representations of ferri-Cyt.*c* (A; PDB 3O1Y), ferro-Cyt.*c* (B; PDB 3O20), Cyt.*c* portion of yeast Cyt.*bc*$_1$–Cyt.*c* complex (C; PDB 3CX5), yeast iso-1 Cyt.*c* (D; PDB 1YCC), Cyt.*c* portion of *Leishmania major* Cyt.*c*–CcP complex (E; PDB 4GED), and *Leishmania major* Cyt.*c* (F; PDB 4DY9). All structures are viewed from the same direction. The *B*-factor color scale is provided below in Å^2 unit. Heme *c* atoms of each Cyt.*c* have lower *B*-factors than those of peripheral residues.

 © 2016 The Authors
